# Development of Glioblastoma from Stem Cells to a Full-Fledged Tumor

**DOI:** 10.5146/tjpath.2022.01582

**Published:** 2023-05-15

**Authors:** Pavel Vladimirovich Nikitin, Guzel Railevna Musina, Valery Nikolaevich Polozov, Dmitry Nikolaevich Goreiko, Vladimir Mikhailovich Krasnovsky, Leonard Werkenbark, Mauric Kjelin, Piotr Sergeevich Timashev

**Affiliations:** Institute for Regenerative Medicine, Sechenov First Moscow State Medical University, Moscow, Russia; Federal Center for Brain and Neurotechnologies, Moscow, Russia; A.A. Bogomolets National Medical University, Kyiv, Ukraine; Kharkiv National Medical University, Kharkiv, Ukraine; N.N. Blokhin Cancer Research Center, Moscow, Russia; University of Brussels, Brussels, Belgium; University of Bordeaux, Bordeaux, France; Institute for Regenerative Medicine, Sechenov First Moscow State Medical University, Moscow, Russia; Chemistry Department, Lomonosov Moscow State University, Moscow, Russia; World-Class Research Center “Digital biodesign and personalized healthcare,” Sechenov First Moscow State Medical University, Moscow, Russia

**Keywords:** Glioblastoma, Carcinogenesis, Intratumoral heterogeneity, Glioma stem cells, Tumor evolution

## Abstract

***Objective:*** IDH wild-type glioblastomas (GBM) are one of the most malignant and complex tumors for treatment. The urgent question of new therapeutic and diagnostic tools searching should be resolved based on cellular and molecular pathogenesis mechanisms, which remain insufficiently studied. In this study, we aimed to investigate GBM pathogenesis.

***Material and Method:*** Using the isolation of different GBM cell populations and the cell cultures, animal models, and molecular genetic methods, we tried to clarify the picture of GBM pathogenesis by constructing a projection from different glioma stem cells types to an integral neoplasm.

***Results:*** We have shown a potential transformation pathway for both glioma stem cells and four definitive cell populations during gliomagenesis. Moreover, we have characterized each population, taking into account its place in the pathogenetic continuum, with a description of the most fundamental molecular and functional properties.

***Conclusion:*** Finally, we have formed a complex holistic concept of the pathogenetic evolution of GBM at the cell-population level by integrating our results with the data of the world literature.

## INTRODUCTION

Glioblastoma, IDH-wild type (GBM), is one of the deadliest human tumors, and the existing treatment options leave much to be desired (1,2)). The one-year overall survival of patients with GBM averages 40.2%, while the five-year overall survival is 5.6% (1,2). So, the existing approaches are insufficient to achieve good results in the treatment of GBM and need to be improved (3,4). At the same time, it is evident that the development of new therapeutic tools should be based on progress in understanding the nature of the disease and its pathogenesis (3,4). Tracing the pathways of GBM development at different stages of carcinogenesis sheds light on the internal mechanics of the pathological process, allowing the creation of tools for a point diagnostic application and targeted treatment (3–6). Such a precision personalized approach with deep molecular and pathogenetic rationale can significantly improve the efficiency of medical care for GBM patients.

Previous research showed that *neural stem cells* are the probable *locus origins* of GBM and other diffuse gliomas (7,8). It is most likely that they are the original ancestors of the primary tumor clone, which later gives rise to a full-fledged tumor node. The nature of normal stem cells’ carcinogenetic transformation remains a mystery, possible solutions to which are spontaneous mutations and mistakes during possible rearrangements of the neuronal stem cells genome during brain plastic rearrangements (9,10). An important role here is played by the features of the earliest mutational events, which predetermine the type of the future tumor through the molecular profile of the early precursors of glial neoplasm. In the case of *IDH1*/*IDH2* mutations, further development of the tumor population is directed towards diffuse astrocytoma, anaplastic astrocytoma, or oligodendroglial tumors, while *EGFR*, *CDKN2A*/*B*, and *PTEN* mutations become early driver events for the GBM (11,12).

The principal reflection of the carcinogenesis mechanisms and their product is heterogeneous tumor cell populations in their molecular and functional properties. A necessary type of such population is the very early precursors of glial neoplasm, which persist at later stages as a component of an integral large tumor node as glioma stem cells (GSC) (13,14). There are two principal types of GSC – proneuronal GSC (PGSC), which demonstrate high expression of neuronal differentiation and neuronal stem cells genes, as well as mesenchymal GSC (MesGSC), in the transcriptional profile of which, along with neuronal genes, mesenchymal differentiation genes predominate (13,14). These types of GSC largely determine the characteristic features of the neoplasm biological behavior and the degree of its aggressiveness. In particular, MesGSC provides tumor insensitivity to therapeutic and radiation treatment (13–16). Both GSC variants are the source of the formation of the initial tumor clone and the source of the current repopulation of the entire cell array (13–16).

There are also definitive cell populations within GBM, isolated using transcriptional single-cell profiling, reflecting several features and axial patterns of the pathogenetic process at its later stages (17). Neftel et al. showed that tumor cells in GBM can be in four primary definitive cellular states (18,19). These states resemble in their molecular profile different types of cells in the nervous system. Among them, neural progenitor-like (NPC-like), oligodendrocyte-progenitor-like (OPC-like), astrocyte-like (AC-like), and mesenchymal-like (MES-like) states were found. Moreover, in each of these states, characteristic molecular features that serve as drivers for the cell population were identified. For NPC-like, this feature is *CDK4* gene amplification, OPC-like *PDGFRA* gene amplification, AC-like *EGFR* gene amplification, and MES-like Chr5q deletion or *NF1* mutations, each of which contributes to the development of a particular cellular state. In addition, it turned out that some of the cells are in a hybrid state that combines the two molecular profiles described above. It is also interesting to note that two cell states or populations – MES-like and NPC-like – were additionally quite clearly subdivided into two more subpopulations – MES1 with increased expression of the *DDIT3* gene and MES2 with increased expression of the *ANXA2* gene in the MES-like cell population, as well as NPC -like 1 with increased expression of the *DLL3* gene and NPC-like 2 with a high level of expression of the *STMN2* gene in the NPC-like cell population (17–20).

In the framework of previous studies, we examined the issues of intratumoral heterogeneity in a slightly different context, demonstrating the heterogeneity of the distribution of tumor cells’ functional activity depending on the position of these cells concerning key histopathological landmarks – tumor vessels and necrosis. The critical functions of tumor cells taken as the basis for the analysis were proliferative and antiapoptotic activity. As a result, we identified five cell clusters with different features and properties (18).

Thus, even though some aspects of different stages of the GBM carcinogenetic pathway have been well studied to a certain extent, a holistic, fundamental understanding of the pathogenetic process has not been formed. Within the framework of this study, we set the task of studying several features of both early and later stages of GBM development, projection from different GSC variants through their functional aspects at different stages of tumor node formation to a full-fledged neoplasm containing, in addition to stem cells, all four critical definitive cell populations. At the same time, to create the complete picture, an integrated approach is used, combining the assessment of not only functional but also molecular and pathohistological properties of tumor cells, including their location in a particular histological niche or histological zone.

## MATERIAL and METHODS

### General Description of the Study

The study included samples of 48 intracerebral tumors obtained during the neurosurgical intervention in the Federal Center for Brain and Neurotechnology and the Burdenko Neurosurgical Institute in 2014 – 2016. The ethics committee approved this study of the Federal Center for Brain and Neurotechnology and the Burdenko Neurosurgical Institute. Patients included in the study signed informed consent to participate in the study. According to the histopathological examination results, all tumors included in the study were verified by three experienced neuropathologists and to molecular typing as GBM, IDH-wild type. Inclusion criteria were age over 18 years, primary surgery for this neoplasm, lack of previous treatment, including chemotherapy and radiation therapy, and the presence of a single neoplasm at the time of surgery. If patients did not meet these criteria, we excluded them from the study.

The average age of the patients was 58.24±4.42 years. The number of men was slightly higher than the number of women – 56.73% and 43.27%, respectively. In 44 cases, the tumor was removed, and in 4 cases, stereotactic biopsy took place. In 29.17% of cases, the tumor was located in the temporal lobe, 25% cases in the parietal lobe, 25% cases in the frontal lobe, and 20.83% cases in the occipital lobe.

All experimental procedures, which included human-derived objects, fully complied with the principles of the Declaration of Helsinki and were approved by the local ethics committee of Sechenov University. All manipulations during experiments using animal models were complied with the ARRIVE guidelines and were carried out in accordance with the U.K. Animals (Scientific Procedures) Act of 1986 and EU Directive 2010/63/EU for animal experiments.

### Pathohistologic Examination

After description and placement of tumor fragments in histological cassettes, the material was fixed in formalin (Sigma-Aldrich, USA), and the blocks were guided through alcohols for dehydration and degreasing, and the blocks were paraffin impregnated. Then, we cut 3-micrometer sections from the blocks on a microtome. After that, the paraffin was removed from the sections in two xylenes, two minutes each. The slides were washed from xylene in two absolute alcohols for 1 minute and two 96C° alcohols for 1 minute. Then slides were washed in water and dipped in hematoxylin to stain the cell nuclei for 5 minutes (Mayer’s hematoxylin, Sigma-Aldrich, USA). After hematoxylin, slides were washed with water to remove excess dye. Then the slides were immersed in 5% eosin (Sigma-Aldrich, USA) to stain the cell cytoplasm for 30 seconds and again washed with water to remove excess dye. After that, slides were dehydrated in two 96C° alcohols for one minute and clarified in a solution of carbol-xylene. Then the slide was washed from carbol-xylene in xylene. The section was placed under a cover glass.

The samples obtained after placing various types of GSC into cell cultures and their subsequent incubation under different conditions were also subjected to processing, fixation in paraffin, and subsequent staining similarly to the described method.

### Flow Cytometry

We used flow cytometry to solve the problem of sorting different types of tumor stem cells and their separation from other populations of tumor and non-tumor cells. In the beginning, GBM tissue obtained from patients in the form of an 8.0-mm section was dissected and placed in the digestion buffer in the form of a solution of RPMI and Accumax at a ratio of 1:1 (Thermo Fisher Scientific, USA). Tissue in 30% Percoll’s solution diluted in RPMI (Thermo Fisher Scientific, USA) was wiped through a mesh with a diameter of 70 μm. Then, all the cells were centrifuged for 15 min at 10°C at a speed of 1500 G to precipitate them. After that, we suspended the tumor cells in a buffer for lysis of erythrocytes; then, they were precipitated. Next, the obtained cell mass was washed with PBS and suspended in a buffer using antibodies for the other labeling process.

Then, endogenous Fc antibody fragments were blocked for 15 min under cooling conditions using CD16 and CD32 neutralizing antibodies at a dilution of 1:100 (Sigma-Aldrich, USA). The GBM cells obtained at the previous stage were washed and resuspended in an antibody cocktail containing antibodies to specific MesGSC markers CD109 (Invitrogen, Thermo Fisher Scientific, USA), Lyn (Invitrogen, Thermo Fisher Scientific, USA), and WT1 (Invitrogen, Thermo Fisher Scientific, USA), as well as PGSC markers CD133 (Invitrogen, Thermo Fisher Scientific, USA), Sox2 (Invitrogen, Thermo Fisher Scientific, USA) and Notch1 (Invitrogen, Thermo Fisher Scientific, USA), which were incubated with cells for one h on ice before washing and resuspending in cell staining buffer.

We applied fluorescence-activated cell sorting (FACS) (iCyt sy3200, Sony Biotechnologies, USA) to isolate MesGSC and PGSC from all types of cells, and for separate sorting. Flowjo (v. 10.6.2, USA) was used as the software for analysis. We based the gating process on the analysis of forwarding and side scatter plots to isolate the cell from debris and exclude possible doublets. In addition, this assay allowed compensation using single antibody controls. Single-channel, unstained experimental variants, and fluorescence minus one were used as controls. MesGSC (CD109+/ Lyn+/ WT1+/CD133-/Sox2-/ Notch1-) and PGSC (CD133+/Sox2+/ Notch1+/ CD109-/ Lyn-/ WT1-) were identified and sorted into separate tubes.

### Cell Cultures

**Figure 1:**
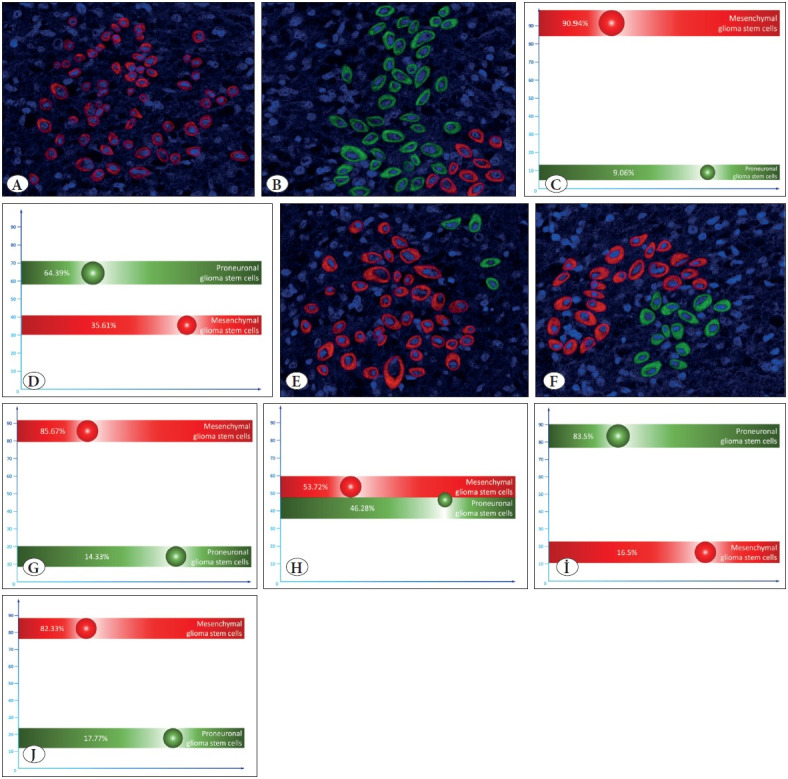
**A,B)** Show the results of immunofluorescent staining to identify markers of mesenchymal glioma stem cells CD109 (red marks) and proneuronal glioma stem cells CD133 (green marks) visualized by confocal microscopy in the organotypic adult brain hemisphere slice culture with mesenchymal and proneuronal patient glioma stem cells inoculation respectively. **C,D)** illustrate the average percentage of mesenchymal and proneuronal glioma stem cells after 8 hours of incubation of organotypic brain slice culture with mesenchymal and proneuronal glioma stem cells, respectively. From now on, the mean values of the indicators on the graphs correspond to the circle’s white center, and the circle’s diameter corresponds to the indicator’s ±σ. The results of immunofluorescent staining for the detection of markers of mesenchymal glioma stem cells CD109 (red marks) and proneuronal glioma stem cells CD133 (green marks) with confocal microscopy visualization in the organotypic rat subventricular slice culture after mesenchymal **(E)** and proneuronal **(F)** glioma stem cells inoculation and 24 hours incubation are shown in Figures **E,F** respectively. For cases with mesenchymal **(G)** and proneuronal **(H)** glioma stem cells inoculation, the results of the content percentage counting for each glioma stem cell type are shown in Figures **G,H,** respectively. **I,J)** show the results of the percentage calculation of mesenchymal and proneuronal glioma stem cells after incubation of tumor cell cultures under conditions of relative normoxia **(I)** and hypoxia **(J).**

C57BL/6m mice at the age of 5 – 6 weeks were used to create different cell cultures. At the first stage, the brain was removed from the mice, after which we placed the brain in a tissue culture dish containing sterile PBS solution and placed it on ice (Figure 1). Within the framework of this study, two types of organotypic slice brain culture were obtained, including *hemisphere slice culture* and *subventricular slice culture* at the subsequent stages. To create a hemisphere slice culture, the mouse forebrain was isolated and placed in a dish with preheated 3% SeaPlaque agarose (Lonza Bioscience, Switzerland); the rest of the brain, including the cerebellum, brain stem, and olfactory bulbs, was removed. To isolate the subventricular slice culture, the subventricular zone of the mouse brain tissue was excised, and then placed in a dish with preheated 3% SeaPlaque agarose (Lonza Bioscience, Switzerland); we removed the rest of the brain. The material cooled with ice was cut with a scalpel into small cubic pieces with a facet size of 2 cm. Then a six-well Millicell Cell Culture Insert plate (Merck, USA) was used, into the wells of which one cell culture fragment was injected together with 1 ml of culture medium diluted in serum-free primary NSC medium, Dulbecco’s modified Eagle medium (Life Technologies, Thermo Fisher Scientific, USA). An embedded brain was placed in a Leica VT1200S vibratome plate (Leica, Germany) fixed on a platform using a special glue, after which the platform was filled with PBS with penicillin-streptomycin at a dilution of 1:100 (Thermo Fisher Scientific, USA). After that, sections with 250 μm thickness were made with a vibration frequency of 8 and a speed of 3. Then each section was transferred to the upper part of the Millipore culture insert. We placed a six-well plate in an incubator, and then the incubation process took place at 37°C, 5% CO2, and the sections were incubated for 24 – 48 hours before tumor cell transplantation.

### Seeding GSC into Cell Cultures

We applied a Gilson pipette to transplant MesGSC and PGSC isolated from human GBM tissue with flow cytometry; using a pipette, 0.2 μL of cells were added to the center of the incubated cell culture. Before implantation, centrifuged cells were harvested and resuspended in solution to a concentration of 100 000 cells/μl. We used an automatic nanoliter injector (Nanoject II, Drummond Scientific, USA) to inject 40 nL of GSC. All injections were performed using tips with a diameter of 10 – 20 μm and an automatic needle remover (Drummond Scientific, USA). In a single injection, we injected 200 nL into a socket in a section of the cell medium made with forceps to facilitate the engraftment of GSCs in the medium. With a single injection, 4000 GSC were transplanted. We incubated cell cultures with GSC under different conditions depending on the experimental objectives. As part of the simulation of classical conditions of tumor growth, cell cultures were incubated at 37°C and 3% O2 for 1 and 8 days; in the case of simulating hypoxic conditions, we incubated the cell cultures at 37°C and 1% O2 for one day. Fresh StemPro NSC SFM medium (Thermo Fisher Scientific, USA) was added every two days. We monitored the growth of the planted cells in the cell medium using a Leica M205 C stereomicroscope (Leica, Germany), and the engraftment of cells was observed 3 – 4 days after transplantation.

After incubation under different experimental conditions, tumor nodes grown in cell cultures were excised, sectioned, and stained.

### Seeding GSC in the Mouse Brain

MesGSC and PGSC isolated from GBM patient’s samples by flow cytometry were plated in agar-coated flasks (0.85%) and allowed to self-organize and form organoids for about two weeks at 37°C and 5% CO2 in DMEM medium containing 0.4 mM NEAA, two mM L-glutamine, 10% FBS, and 100 U/ml Pen-Strep (Lonza Bioscience, Switzerland). After that, the obtained organоids, which had a diameter of 400 – 900 μm, were implanted into the brains of nude mice (NOD/Scid) using a Hamilton syringe (Hamilton, USA) into the right frontal cortex. The animals were kept under SPF conditions and sacrificed depending on the experimental design 6 or 8 weeks after implantation. Tumor volume was monitored using MRI. Further, according to the visual macroscopic assessment, we took the material to include the tumor zone and peritumoral fragments of brain tissue. After that, we subjected the material to pathohistological examination with hematoxylin and eosin staining according to the method described above in all cases and, depending on the task of the experiment, to confocal microscopy multiparametric fluorescent *in situ* hybridization (FISH).

### Tissue Staining

The GBM samples obtained at the previous stages were incubated with primary mouse monoclonal antibodies to CD133 (Thermo Fisher Scientific, USA) at a dilution of 1:100 and primary rabbit monoclonal antibodies to CD109 (Thermo Fisher Scientific, USA) at a dilution of 1:100 for 24 hours at 4°C, followed by secondary antibodies conjugated with Alexa Fluor 488 dye (Thermo Fisher Scientific, USA) at a dilution of 1:100 for CD133 and secondary antibodies conjugated with Alexa Fluor 594 dye (Thermo Fisher Scientific, USA), at a dilution 1:100 for CD109 for 1 hour at room temperature. After each staining period, two 15 minutes washes were performed with PBS. To stain the nuclei, the samples were incubated with DAPI dye (Thermo Fisher Scientific, USA) at a dilution of 1:10 000 in PBS buffer for 1 hour. At the next stage aiming to carry out microscopy on glass slides, vaseline strips with a height of 4 mm were prepared with the further addition of the mounting medium Fluoromount-G (Thermo Fisher Scientific, USA), into which we placed the washed samples. Then a unique glass lid was attached on top; the edges were sealed with a transparent varnish.

### Confocal Microscopy

A Leica STELLARIS confocal microscope (Leica, Germany) was used for laser scanning confocal microscopy. In the process of image obtaining, in all cases with a standard layer thickness of 30 μm, shooting was carried out with a step of 4.5 μm between layers. We used the ImageJ (NIH, USA) and Imaris (Bitplane, Switzerland) software for the volumetric rendering of z-stacks in three-dimensional space (21). Further, the threshold values were established for the fluorescent labels’ volume, ellipticity, and signal quality for better differentiation of individual cells of the neoplasm. Then, using ImageJ and Imaris, the quantitative assessment of images was carried out to determine the percentage of cells with specific marker positive expression and the analysis of the spatial position of tumor cells, primarily relative to the vessels’ walls and necrosis (22).

### Multiparametric FISH

The prepared tumor sections, placed on glass slides and frozen at –80°C, were quickly transferred into a pre-cooled 4% paraformaldehyde solution (Sigma-Aldrich, USA). Further, we incubated all samples for 15 minutes at 4°C. After that, slides were washed in 1xPBS solution at room temperature five times for 2 min. Then slides were dehydrated in 50% ethanol, 70% ethanol for 5 minutes, and twice in fresh 100% ethanol at room temperature. After excess liquid removal, the slides were dried in the air on a flat surface for 5 minutes at room temperature. Then slides were wholly immersed in a solution for pretreatment with protease IV (Advanced Cell Diagnostics, Bio-Techne, USA) and incubated for 30 minutes at room temperature. RNA probes complementary to the *DDIT3*, *ANXA2*, *EGFR*, *PDGFRA*, *DLL3*, and *STMN2* messenger RNAs were used (Advanced Cell Diagnostics Bio-Techne, USA). *DDIT3* was marked blue, *ANXA2* was green, *EGFR* was yellow, *PDGFRA* was orange, *DLL3* was red, and *STMN2* was dark red. To prepare the probes, we preheated them for 10 min at 40°C in a water bath. A mixture of RNA probes was prepared in tubes without RNase content. The concentration of each probe in the solution was 1:50. Then the slides were transferred from the pretreatment solution to the 1xPBS solution at room temperature, washing them and incubating them twice for 2 minutes. After that, we transferred slides to a horizontal slide rack in a preheated humidifying chamber, and 50 μL of the probe mixture was pipetted onto each slide.

Next, probe hybridization was carried out for 2 hours at 40°C in a sealed humidified chamber. After that, we removed the hybridization solution excess from each slide and treated it with 1x wash buffer at room temperature for 2 minutes twice. Excess 1x wash buffer was then removed from each slide, and the slides were transferred to a humidified chamber. All slides were covered with amplification solution AMP1 (Advanced Cell Diagnostics, Bio-Techne, USA) and incubated in a humidified chamber for 30 minutes at 40°C. Afterwards, all slides were washed twice in 1x wash buffer at room temperature. The amplification solution AMP2 (Advanced Cell Diagnostics, Bio-Techne, USA) was then added and incubated for 15 minutes at 40°C, followed by washing all slides twice in 1x wash buffer at room temperature. At the next stage, amplification solution AMP3 (Advanced Cell Diagnostics, Bio-Techne, USA) was added and incubated for 30 minutes at 40°C, followed by washing the slides twice in 1x wash buffer at room temperature. Finally, Amplification solution AMP4 (Advanced Cell Diagnostics, Bio-Techne, USA) was added and incubated for 15 minutes at 40°C. Then the slides were washed twice in 1x wash buffer at room temperature. This was followed by staining of cell nuclei with the addition of 2 drops of DAPI (Thermo Fisher Scientific, USA) and incubation for 30 seconds at room temperature. At the next stage, we added 10 μL of Mowiol DABCO aqueous mounting medium (Thermo Fisher Scientific, USA) to each slide, and a coverslip was placed on each slide. Next, the slides were placed in a dark room for 12 hours at 4°C to dry completely.

All slides were then subjected to confocal microscopy, followed by quantitative assessment of signals from all studied messenger RNAs using ImageJ and Imaris software.

### Statistical Analysis

The SPSS Statistics 26.0 software (IBM, USA) was used to carry out a statistical analysis of the obtained results. We carried out intergroup comparisons using the Mann-Whitney U-test in the case of abnormal distribution of the trait, and Student’s t-test in the case of a normal distribution of the trait. The character of the distribution of the feature was determined using the Shapiro-Wilk test. The influence of the studied factors on patient survival was determined using the Cox proportional hazards model. For greater clarity of the dependence of the overall survival on the tumor cell-population composition, the Kaplan-Meier curves were plotted. For this, we divided all the cases into two groups – with a high and a low level of quantitative parameters under consideration. The average value of the corresponding parameter was determined, and each case was then ranked into one of the groups – into the high group, if the parameter, in this case, is higher than the average, and into the low group if the parameter, in this case, is below the average. Differences were considered significant at p<*0.05*.

## RESULTS

### Early Stages of Pathogenesis

By sorting cells using flow cytometry, we isolated MesGSC and PGSC from tumor samples of 48 patients with an established diagnosis of GBM, IDH-wild type, after which we separately placed MesGSC and PGSC in organotypic adult brain hemisphere slice culture. After incubation in these cultures for 8 hours, it was shown that the number of tumor cells was, on average, higher in cultures with MesGSC overseeding than in cultures with initial PGSC overseeding (Figure 1), and the difference was statistically significant (p=0.034). Next, we evaluated how the qualitative composition of GSC in these cultures changed after 8 hours of incubation using an immunofluorescence study. It turned out that if in the culture with MesGSC inoculation, there is an exclusive presence of MesGSC (n = 42), or their significant predominance (n=6) in the overwhelming majority of cases after 24 hours. In the culture with PGSC inoculation, the cell composition is mixed; however, on average, the prevalence of PGSC is revealed (Figure 1).

In order to further study the early stages of gliomas development, we placed the MesGSC and PGSC isolated from all 48 patients separately in the organotypic rat subventricular slice culture to create the most realistic model of the potential possible tissue environment for the early stages of GBM development. In this case, we also obtained interesting results, repeating those previously stated in the paragraph above. After incubation for 24 hours, the number of tumor cells in cultures with initial MesGSC inoculation exceeded that in cultures with PGSC inoculation with statistical significance (p=0.025). When typing tumor cells in cultures with MesGSC inoculation, the predominance of MesGSC was also observed, but already slightly less significant, since MesGSC was observed exclusively only in 34 cases; in the rest, this type of cell only prevailed. A predominance of MesGSC on average was also observed in cultures with PGSC overseeding, but it was not as significant as in the case of MesGSC inoculation (Figure 1).

We assumed that the observed results might be due to the characteristics of the environment and the content of oxygen and nutrients in it. In this regard, we re-seeded PGSCs (n=48) in the organotypic adult brain hemisphere slice cultures and placed them in an incubator at different O2 levels (23). We showed that under normal conditions when the O2 level is 3%, which corresponds to the oxygen content in the peritumoral brain tissue according to studies, there is a significant predominance of PGSC with only a tiny amount of MesGSC after 24 h incubation (Figure 1). At the same time, incubation of similar cell cultures at an O2 level of 1% showed a significant predominance of MesGSC in all cases (Figure 1). The differences were statistically significant (p<0.001 and p<0.001).

Thus, at the early stages of gliomagenesis, the predominance of MesGSC is most likely, which can be explained by a high degree of competition for nutrients and oxygen, and, consequently, their relatively low availability at this stage of neoplasm development. In this regard, switching to the mesenchymal phenotype of stem cells seems to be an adaptive response that allows glioma-initiating cells to survive at an early stage of pathogenesis. At the same time, it is possible that the initially emerging tumor clone can be represented as PGSCs, which then switch to MesGSC in a competitive environment, and initially this clone can be represented by MesGSC.

### GSC in the Formation of a Tumor Node in Slice Culture

**Figure 2:**
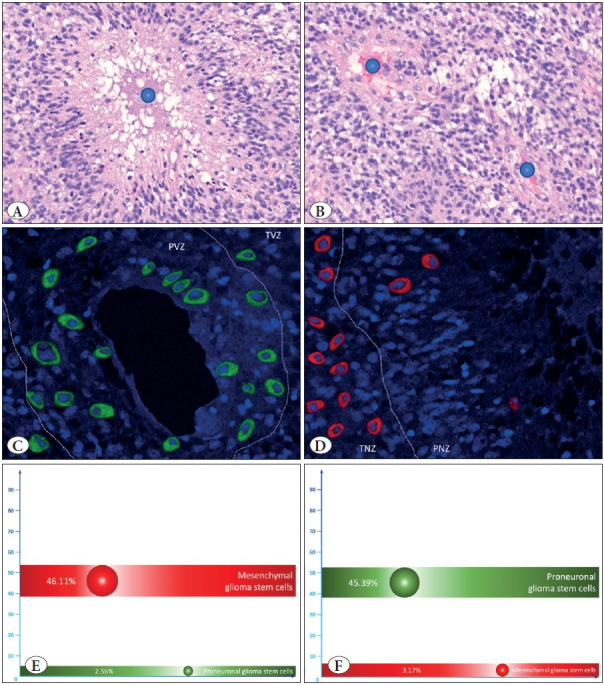
**A,B)** Show the pathohistological picture of glioblastoma grown after mesenchymal glioma stem cells inoculation in the organotypic adult brain hemisphere slice culture and incubation for eight days. Crucial pathohistological characteristics - necrosis **(A)** and proliferation of the vascular endothelium **(B)** - are presented. Figures **C,D** show the results of immunofluorescent staining to identify markers of mesenchymal glioma stem cells CD109 (red marks) and proneuronal glioma stem cells CD133 (green marks) with confocal microscopy visualization in the area of necrosis **(C)** and tumor vessels **(D)**. Figures **E,F** show the average percentage of mesenchymal and proneuronal glioma stem cells in the perinecrotic **(E)** and perivascular **(F)** zones.

Next, we seeded MesGSC from all 48 patients on organotypic adult brain hemisphere slice cultures to study the further nature of changes in different types of GSC. After eight days of incubation, we found the formation of macroscopically detectable tumor nodes in the overwhelming majority of cases (n = 44). We carried out a histological examination of all slice cultures. We found typical histological gliomas in 42 cases with the presence of highly malignant tumor cells forming different histological structures and the essential histological criteria of GBM in the form of necrosis (Figure 2) and proliferation of the vascular endothelium (Figure 2).

Then we studied the qualitative and quantitative distribution of MesGSC and PGSC in different zones in GBM tissue obtained from slice culture. Using immunofluorescence and confocal microscopy, we identified the characteristic patterns of distribution of these cells’ types; in particular, we found that most often, PGSCs are found in the area immediately around the vessels (Figure 2), with MesGSC in the area close to necrosis (Figure 2).

In order to quantitatively calculate and confirm this regularity, we conditionally identified several pathohistological zones in the tumor tissue. We identified the perivascular zone (PVZ); to conventionally outline its boundaries, we empirically determined them at a distance of five cells from the vascular wall in all directions (Figure 2). Around this zone, we empirically identified a transient vascular zone (TVZ) for ten more cells. We have drawn similar conditional zones around necrosis. Directly around the necrotic detritus, we identified a perinecrotic zone (PNZ) extending from the necrotic zone by ten cells in each direction (Figure 2). Further, for another 20 cells, we outlined a transient necrotic zone (TNZ). The rest of the tumor areas outside the above zones were conditionally included in the intermediate zone (IZ).

Quantitatively, we have shown that the content of MesGSC has the highest precision in the TNZ, in which the content of PGSC practically tends to zero (Figure 2). At the same time, the most significant number of PGSCs is concentrated in the PVZ, within which MesGSCs practically do not occur (Figure 2). The differences in the content of the considered cell types in both TNZ and PVZ were statistically significant (p<0.001 for both cases).

### Distribution of GSC Types in the Nude Mice Model

**Figure 3:**
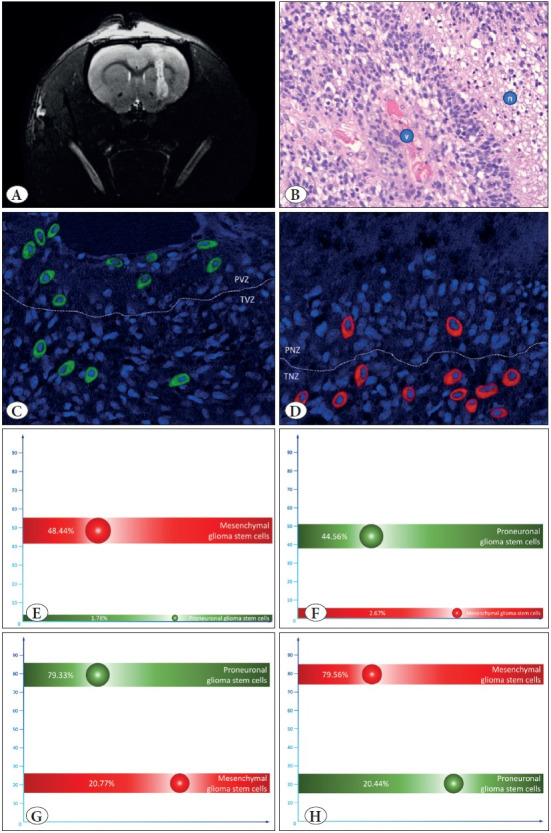
**A)** Shows magnetic resonance imaging of a nude mouse brain in T2 mode six weeks after implantation of mesenchymal glioma stem cells culture. The corresponding histopathological picture is shown in **(B);** it clearly shows the classic signs of glioblastoma, in particular the zone of necrosis (marked with the letter “n”) and the zone of the vascular endothelium proliferation (marked with the letter “v”). **(C,D)** show the typical distribution of mesenchymal and proneuronal glioma stem cells in the perivascular zone (PVZ) and transient vascular zone (TVZ) **(C),** as well as the perinecrotic zone (PNZ) and transient necrotic zone (TNZ) **(D)**. In this case, we performed immunofluorescent staining with visualization by confocal microscopy to identify markers of mesenchymal glioma stem cells CD109 (red marks) and proneuronal glioma stem cells CD133 (green marks). **(E,F)** show the mean percentages of mesenchymal and proneuronal glioma stem cells in TNZ **(E)** and PVZ **(F).** **(G)** shows the average percentage of different glioma stem cells in organotypic slice culture after mesenchymal glioma stem cells inoculation and incubation under normoxic conditions. (H) shows the average percentage of different glioma stem cells in organotypic slice culture after implantation of proneuronal glioma stem cells and incubation under hypoxic conditions.

Next, we decided to confirm our findings using the nude mice model. For this purpose, we randomly selected 38 MesGSC samples from different patients and transplanted them into the brains of MesGSC nude mice. After six weeks of incubation, we performed an MRI scan on all mice to confirm sufficient tumor growth (Figure 3). All animals survived to 6 weeks, and then samples of the grown tumors were taken from them. We initially subjected all tumor samples to histopathological examination. As a result, we identified a typical pathological picture of GBM with characteristic features (Figure 3).

To identify MesGSC and PGSC markers, we carried out an immunofluorescent study with confocal microscopy and revealed a similar picture with slice culture. It has been shown that in the PVZ, PGSCs are dominant (Figure 3). In addition, the prevalence of MesGSC in the TNZ was revealed, also mainly found in this zone (Figure 3). A quantitative calculation confirmed these suggestions. MesGSC made up a significant proportion of cells in the TNZ, with an almost complete absence of PGSCs markers in this zone (Figure 3). At the same time, PGSCs were quantifiable in the PVZ with a negligible amount of MesGSCs in the zone (Figure 3). These differences in TNZ and PVZ were statistically significant in both cases considered (p<0.001 for both cases).

Then we decided to study the features of changes in different types of stem cells in GBM. For this, we carried out sorting of MesGSC and PGSC from tumors previously excised in mice using flow cytofluorometry. We seeded these stem cells types separately from all 38 animals on primary organotypic slice cultures and incubated them for 24 hours under different O2 conditions. Considering the previous results, we decided to incubate primary organotypic slice cultures with MesGSC cells in an environment with an increased O2 content of up to 3%, but PGSC, on the contrary, under a sharply reduced condition O2 content to 1%. As a result, we found that in the culture with the initial over-seeding of MesGSC, kept under normal oxygen partial pressure, there was a significant predominance of PGSC (Figure 3). At the same time, a pronounced predominance of MesGSC was revealed in the culture with PGSC overseeding at the beginning, which was in hypoxic conditions (Figure 3). Moreover, the differences revealed in both cases were statistically significant (p=0.007 and p=0.005).

Thus, we have demonstrated the presence of histopathological zones typical for different types of stem cells and showed the pathogenetic mechanisms of the formation of these stem pools. Their appearance is directly related to the availability of nutrients and oxygen and the dependent switching of stem phenotypes. When unfavorable hypoxic conditions are formed, a switch to MesGSC occurs, while good oxygen and nutrient conditions contribute to the transition to the PGSC stem subtype.

### Different Cell Populations

**Figure 4:**
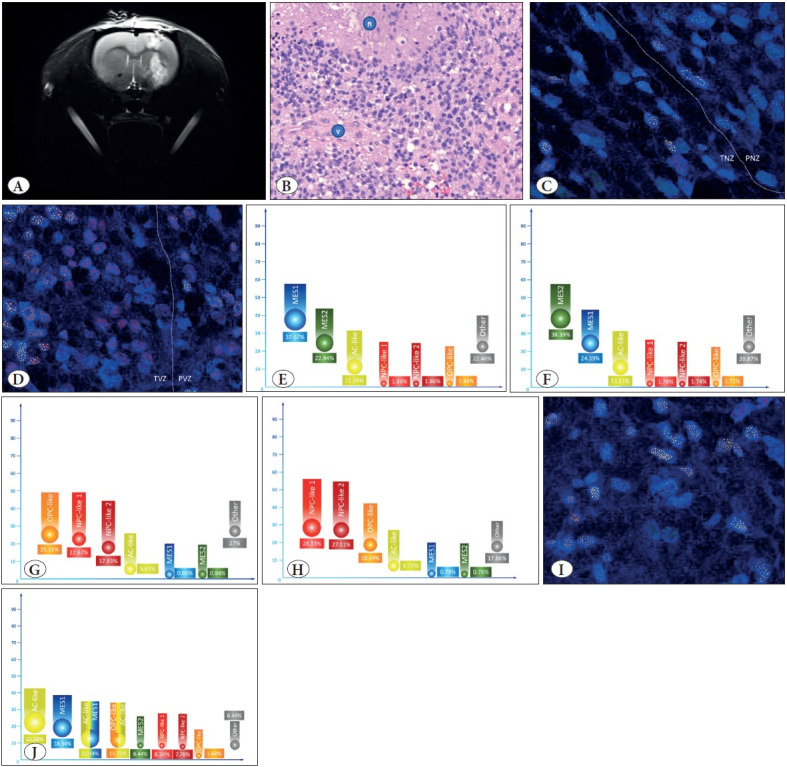
**A)** shows magnetic resonance imaging of the nude mouse brain in T2 mode eight weeks after mesenchymal glioma stem cells culture implantation. A typical histological pattern is shown in **(B),** with critical features of glioblastoma, including a zone of necrosis (marked with the letter “n”) and a zone of vascular endothelium proliferation (marked with the letter “v”). **C,D** show a typical pattern of the distribution of primary cell populations in the perinecrotic zone (PNZ) and transient necrotic zone (TNZ) **(C)**, as well as in the perivascular zone (PVZ) and transient vascular zone (TVZ) **(D).** Using multimeric fluorescent in situ hybridization, increased expression of DDIT3 messenger RNA to determine the MES1 cell population (blue label), increased expression of ANXA2 messenger RNA to determine the MES2 cell population (green label), increased expression of EGFR messenger RNA to determine the AC-like cell population (yellow label), increased expression of PDGFRA messenger RNA for OPC-like cell population determination (orange label), increased expression of DLL3 messenger RNA for determination of NPC-like 1 cell population (red label), and increased expression of STMN2 messenger RNA to determine the NPC-like 2 cell population (dark red label) using confocal microscopy. The corresponding colors have coded the percentage of these cell populations in TNZ **(E)**, PNZ **(F)**, PVZ **(G),** and TVZ **(H)**. **(I,J)** show the visualization of the primary marker messenger RNAs for identifying cell populations using multimeric fluorescent in situ hybridization and confocal microscopy in the intermediate zone **(I),** as well as the average percentage of cell populations in this zone, including hybrid cell populations with the expression of several marker messenger RNAs **(J).**

To assess the distribution of different populations of tumor cells in tissue niches of GBM, 38 MesGSC samples from different patients were again randomly selected and implanted into the brains of nude mice. Then we incubated the tumor for eight weeks and performed MRI for all mice to confirm sufficient tumor growth (Figure 4). Thirty-six animals survived eight weeks; after the indicated incubation period, samples of ingrown neoplasms were taken from all surviving mice. At first, all samples underwent a pathohistological examination, which showed a typical pathohistological picture of GBM in all cases (Figure 4), and the samples were advanced to the next stage of work.

Further, for all 36 samples, multiparametric FISH was performed using probes to the messenger RNA DDIT3 to identify the MES1 cell population, ANXA2 to isolate the MES2 cell population, EGFR to determine the AC-like cell population, PDGFRA to detect the OPC-like cell population, DLL3 to detect NPC-like 1 cell population, and STMN2 to identify NPC-like 2 cell population. We found that the MES1 cell population is most common in the TNZ, with this population being predominant in the indicated pathological niche (Figure 4). In PNZ, representatives of the MES2 cell population are most often detected; the bulk of this type of cell is concentrated in PNZ (Figure 4).

Cells from the OPC-like cell population were characterized by preferential localization in the PVZ of the pathohistological niche, in which they were the most frequent cell population (Figure 4). Nevertheless, we detected representatives of the NPC-like 1 cell population with a high frequency in this zone (Figure 4). This type of cell predominates in the neighboring TVZ, where NPC-like 2 cells were also found in slightly smaller numbers, for which this zone is the main one (Figure 4). Finally, in the IZ, a rather heterogeneous picture was observed representing different cell populations, and primarily the AC-like cells predominated (Figure 4). However, there were also significant numbers of representatives of the MES1 cell population, in addition to mixed transitional cell variants in the form of AC-like+MES1 and OPC-like+AC-like cell populations (Figure 4).

### Influence of Cell Populations on Survival

**Figure 5:**
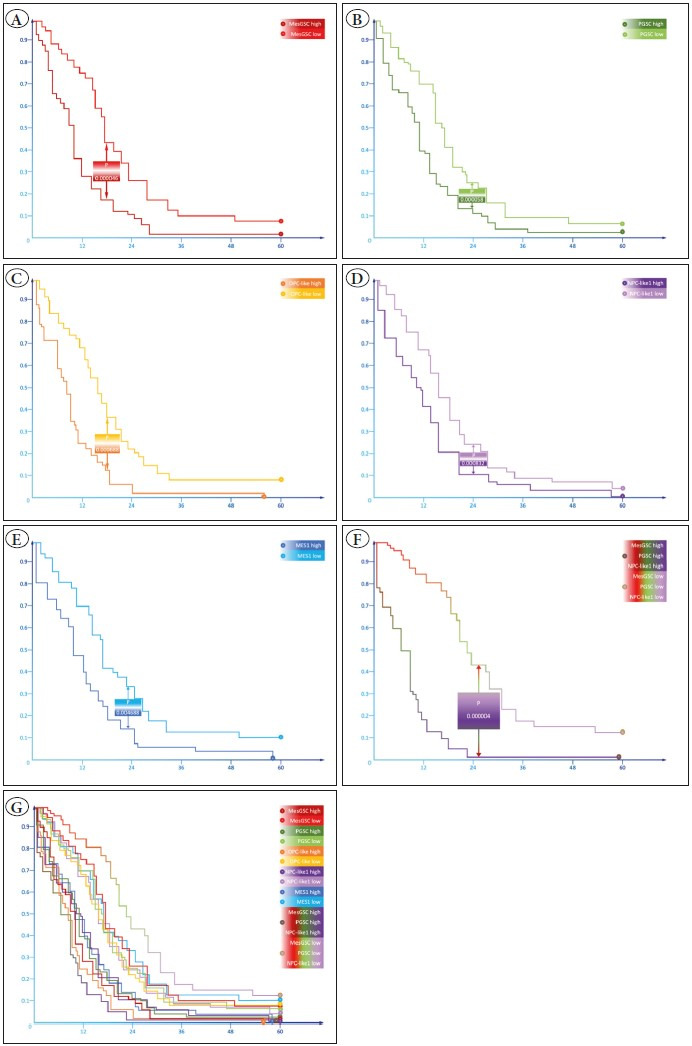
Kaplan-Meier curves of overall survival of patients with high and low levels of mesenchymal glial stem cells in the transient necrotic zone **(A),** proneuronal glial stem cells in the perivascular zone **(B),** OPC-like cell population in the perivascular zone **(C),** NPC-like 1 cell population in the transient vascular zone **(D),** and MES1 cell population in the transient necrotic zone **(E).** In addition, using multimeric regression analysis, it was possible to determine the most prognostically valuable complex of cell-population parameters in the form of a combination of mesenchymal glioma stem cells content in TNZ, proneuronal glioma stem cells content in PVZ, and NPC-like 1 cell population content in TVZ, for which the Kaplan-Meier curves are shown in **(F)**. In addition, all the resulting curves were summarized in **(G).**

Finally, we decided to evaluate the clinical significance of the revealed patterns of different cell populations’ distribution in the tissue niches of GBM. Using regression analysis, we evaluated the effect of the different cell population percentages, including MesGSC, PGSC, MES1, MES2, AC-like, OPC-like, NPC-like 1, and NPC-like 2, on the overall population survival. The content of each cell population was assessed separately in those pathohistological zones where they predominated. It was shown that the content of MesGSC in TNZ (p<0.001), PGSC in PVZ (p<0.001), OPC-like in PVZ (p<0.001), NPC-like 1 in TVZ (p<0.001), and MES1 in TNZ (p=0.005) significantly impact the overall survival (Figure 5). In the multiparameter model, we demonstrated the highest predictive value by the combination of MesGSC content in TNZ, PGSC content in PVZ, and NPC-like 1 in TVZ (p<0.001); the predictive value of this model was the highest among all possible variants (Figure 5).

## DISCUSSION

In this study, we traced the development of GBM from patient-derived glioma stem cells to a full-fledged tumor with typical pathohistological and molecular properties. What is the path that GBM takes until the appearance of clinical manifestations and diagnosis? According to our and published data, MesGSC can act as the initial link in the pathogenetic process. At the initial stage, the development of a tumor occurs in conditions of fierce competition for the necessary plastic and energy substrates between early clones of tumor cells and brain cells. In this regard, the pool of tumor cells is mainly represented by MesGSCs, which are the most resistant to unfavorable metabolic conditions (24,25). Further, MesGSCs gradually spread in the brain tissue from the site of their initial localization, infiltrating the surrounding brain due to the high degree of mobility and the ability to lyse the extracellular matrix, which prevents their free movement (14,15). In addition, MesGSCs can produce angiogenesis factors, stimulating the germination of new blood vessels and, thus, improving their nutrition and making metabolic conditions more favorable for further development (25–28).

The appearance of favorable metabolic shifts leads to shifts in the molecular and functional state, within which MesGSC switches to a different stem profile and becomes PGSC. In conditions of sufficient oxygen and nutrient levels, PGSCs, using, among other things, aerobic glycolysis as the most effective mechanism of catabolism, actively proliferate, giving rise to new cell populations. At the same time, based on the features outlined above, it is not surprising that most cells of this type are localized around the vessels (29,30). The perivascular pool, according to the literature, is not only an ideal metabolic niche that maintains a sufficiently high content of nutrients but is also in a kind of paracrine stimulation connection with the vascular endothelium, which produces factors stimulating stem cell proliferation (for example, NO).

Gradually, with an increase in the number and density of cells in this zone, due to the intensive proliferation of PGSCs, cells supply in the perivascular histological niche deteriorates. On the one hand, this tendency is due to an increased need for nutrients and oxygen because of cell quantity increase. On the other hand, we can also explain it by the peculiarities of tumor vessels in the neoplasm tissue. Newly formed vessels in GBM are defective due to many factors, and, providing up to a specific limit the increasing need of tumor cells for blood supply, reaching the limit of their functional capabilities. They quickly begin to undergo various pathological changes; in particular, there is an increase in endothelial thrombogenicity and the appearance of blood clots in the vessels, which severely disrupt blood flow (31,32). The combination of the considered factors causes a pronounced decrease in the supply of cells with oxygen and nutrients in this zone, a deterioration in the cells’ existence conditions, and acidification of the environment. The latter is an important signal recognized by cells due to the expression of aberrant ion channels ASIC1a and ASIC3 (33,34).

Cells under the most unfavorable metabolic conditions and having the least resistance to hypoxia die and form a zone of necrosis. Cells with a higher resistance, primarily PGSCs, actively begin to move from an unfavorable metabolic zone. At the moment, probably under the influence of negatively changed conditions, the molecular profile of these cells is shifted again, and they are modified into MesGSCs, which can survive better under hypoxic conditions (35,36). An essential role in such a switch can be played by aberrant ion channels, which are likely to act not only as a sensor that increases cell motility but also as a participant in more global molecular shifts up to the transition to a mesenchymal state. MesGSCs then repeat the pathogenetic circle described above, again migrating to more favorable metabolic niches and promoting their creation, producing angiogenesis factors, and then again switching back to the PGSC molecular profile.

The most crucial property of tumor stem cells of both subtypes is their ability to proliferate actively. At the same time, the descendants of these subtypes have a gradually decreasing proliferative potential up to the non-proliferating cell pool, but molecular plasticity is preserved to a certain extent (37,38). This is reflected in the differentiation of the descendants of glioma stem cells into non-stem cells in the four central populations. As mentioned above, one of the most important is MES-like cells, which have a mesenchymal molecular profile and two main subtypes of MES1 and MES2. Cells of both subtypes are, apparently, descendants of MesGSC, while, judging by the data of molecular studies, the differentiation of subtypes occurs under the influence of such a functional factor as hypoxia. The MES1 subtype is hypoxia-independent, while the MES2 subtype shows pronounced signs of a response to hypoxia. Taking into account our data on the localization of cells of both subtypes, it is likely that MesGSCs produce finitely differentiated mesenchymal clones of cells constituting the MES1 subtype during migration already in the zone of relatively favorable metabolic conditions; perhaps, part of the MES1 cells migrate to this zone together with MesGSC. In this zone, which we named TNZ, the metabolic parameters are more favorable in comparison with the tissue site immediately around the necrosis, which we named PNZ; within the TNZ, the effect of hypoxia is not so acute, which is reflected in the molecular properties of MES1 subtype cells and is confirmed by their predominance in this zone. At the same time, some of the MesGSC offspring remain in unfavorable metabolic conditions that have developed in PNZ; their molecular profile demonstrates a pronounced hypoxic component. They form the MES2 subtype, which, according to our data, prevails in the PNZ.

PGSC proliferating under favorable metabolic conditions give rise to NPC-like and OPC-like cell populations. Due to favorable conditions of existence, these cell populations are distinguished by extremely high proliferative activity. The highest proliferation activity is observed precisely in the OPC-like population. At the same time, NPC-like is divided into two subtypes as NPC-like 1 and NPC-like 2. NPC-like 1 is in many ways a transitional subtype between NPC-like and OPC-like cell populations, containing in its molecular profile the features of both populations. At the same time, the proliferative activity of this subtype is exceptionally high and is inferior to that only in the OPC-like population. NPC-like 2 has a greater degree of neuronal differentiation and, although high in general still lower than OPC-like and NPC-like 1 cell proliferation activity (18). The considered data, along with our results, demonstrate that, apparently, the PGSC offspring in the zone of the most favorable metabolic parameters, which develops directly around the vessels and is called PVZ by us, mainly differentiate into an OPC-like cell population.

Nevertheless, in the same PVZ, there is a noticeable proportion of NPC-like 1 cells, which, despite neuronal differentiation, also actively compete for nutrients and oxygen with the OPC-like population, partially acquiring the features of this population. Cells of NPC-like subtype 2 are content with somewhat less favorable conditions in the neighboring TVZ, allowing them to proliferate actively, but to a somewhat lesser extent than cells in PVZ. This pattern is well confirmed by our data, demonstrating the predominance of OPC-like cells in PVZ and the presence of a significant proportion of NPC-like 1, while in TVZ the predominant type was just NPC-like 1.

At the same time, cells in two metabolically favorable tissue zones continue to maintain a certain level of molecular and cellular plasticity. A part of NPC-like cells strives to penetrate from TVZ into PVZ to improve their living conditions. Hence, a mixed cell population is observed, which simultaneously contains OPC-like and NPC-like cell populations. Cell plasticity also manifests itself in the appearance of transitional cell variants, differentiating in the astrocytic direction and forming an AC-like cell population. In this population, cells are present, and can originate from many sources, primarily from MesGSC and PGSC, and probably at the moment when they are close or directly in a state of transition into each other. As a result, tumor cells with molecular signs of astrocytic differentiation are formed, possessing a moderate proliferative activity and, accordingly, located in a zone equidistant from both necrosis and vessels, which we called IZ. It is curious that these cells also retain molecular functional plasticity and various transformations (34). In particular, the increase in the hypoxia zone leads to the mesenchymal molecular program expression in some of the cells of this population and the appearance of hybrid variants between AC-like and MES-like cell populations. Such hybrids can also be associated with the transition from the neighboring zone to more favorable conditions of some of the MES1 subtype cells. At the same time, part of the OPC cells of the cell population, for many reasons, is displaced into less favorable conditions and acquires the features of hybrid differentiation with astrocyte-like molecular traits. In addition, it should be noted that the processes of a phenotypic shift towards the AC-like cell population are widespread not only in IZ but also in TNZ and PVZ, although to a lesser extent.

Thus, in the framework of this study, we tried to model and describe in general terms the carcinogenesis and progression of GBM. Our work emphasizes the importance of the issues of intratumoral heterogeneity since they are the key to understanding the fundamental principles of tumor development. A detailed description of these patterns can become the basis for creating new practical approaches to diagnosing and treating GBM. Our work creates a conceptual basis for further study of the characteristics of cellular and molecular processes of carcinogenesis.

## Conflict of Interest

The authors declare no conflict of interest.

## Funding

World-Class Research Center “Digital biodesign and personalized healthcare,” Sechenov First Moscow State Medical University, Moscow, Russia.

This work was financed by the Ministry of Science and Higher Education of the Russian Federation within the framework of state support for the creation and development of World-Class Research Centers “Digital biodesign and personalized healthcare” №075-15-2020-926.

## Availability of Data and Materials

The datasets used and/or analyzed during the current study are available from the corresponding author on reasonable request.
