# Correction: Development of Glioblastoma from Stem Cells to a Full-Fledged Tumor

**DOI:** 10.5146/tjpath.2023.01598

**Published:** 2023-05-15

**Authors:** Pavel Vladimirovich Nikitin, Guzel Railevna Musina, Valery Nikolaevich Polozov, Dmitry Nikolaevich Goreiko, Vladimir Mikhailovich Krasnovsky, Leonard Werkenbark, Mauric Kjelin, Piotr Sergeevich Timashev

**Affiliations:** Institute for Regenerative Medicine, Sechenov First Moscow State Medical University, Moscow, Russia; Federal Center for Brain and Neurotechnologies, Moscow, Russia; A.A. Bogomolets National Medical University, Kyiv, Ukraine; Kharkiv National Medical University, Kharkiv, Ukraine; N.N. Blokhin Cancer Research Center, Moscow, Russia; University of Brussels, Brussels, Belgium; University of Bordeaux, Bordeaux, France; Institute for Regenerative Medicine, Sechenov First Moscow State Medical University, Moscow, Russia; Chemistry Department, Lomonosov Moscow State University, Moscow, Russia; World-Class Research Center “Digital biodesign and personalized healthcare,” Sechenov First Moscow State Medical University, Moscow, Russia

After the publication of the original article, authors noticed that the authors’ the surnames and patronymics (middle names) had been written in incorrect order. The correct order and spelling of the authors’ names is as belows and the original article has been corrected accordingly.

